# Measuring the Effect of Using a Borderline Students’ Characteristics Model on Reliability of Objective Structured Clinical Examination

**DOI:** 10.7759/cureus.25156

**Published:** 2022-05-20

**Authors:** Rabab A Abed, Shimaa E Elaraby

**Affiliations:** 1 Medical Education, Faculty of Medicine, Suez Canal University, Ismailia, EGY; 2 Medical Education, Fakeeh College for Medical Sciences, Jeddah, SAU

**Keywords:** assessment, bordeline, objective structured clinical exam (osce), standard setting, test reliability

## Abstract

Defining borderline group is a crucial step when applying standard setting methods in objective structured clinical examination (OSCE). However, it is the most challenging and demanding task. This study aimed to measure the effect of using a model describing characteristics of borderline students on the reliability and metrics of OSCE. This model was adopted from a qualitative study based on conducted semi-structured interviews with experienced raters. The model identifies several themes categorized under four items which are gathering patient information, examining patients, communicating with patients, and general personal characteristics. In the current study, two groups of evaluators were investigated: one as the experimental group that received orientation about the used model and the other as the control group that did not receive any orientation. We applied the model in two mirrored OSCE circuits. Using the model enhanced raters' global rating. Consequently, the cut scores between the two OSCE circuits were different, and the examination reliability and quality metrics were improved.

## Introduction

Objective structured clinical examination (OSCE) is an assessment method used to assess students’ clinical performance in a simulated environment. It was first implemented by Harden in 1975, where students moved around 18 stations and two rest stations. In each station, the student is required to perform a specific clinical skill such as clinical examination, history taking, performing procedure, etc. OSCE overcomes the drawbacks encountered in traditional assessment methods such as low validity and reliability of examination, as it has representative samples of required tasks, multiple trained examiners, standardized patient performance, and standardized scoring rubrics [1]. The results obtained from OSCE checklists are used in high stake decisions, thus the examination should be reliable and valid.

Standard setting is one of the methods used to set the expected pass or fail cut score in an OSCE station [2]. Standard setting refers to the process of establishing one or more cut scores on examinations. The cut scores divide the distribution of examinees’ test performances into two or more categories [3].

Standard setting methods are divided into two categories: relative (norm-referenced) and absolute (criterion-referenced) procedures. A relative standard is using the performance of the group of test takers to set standards for each item of the examination. On the other side, the absolute method depends on how many items should be performed correctly to pass [4,5]. Absolute methods are divided into empirical and judgmental methods. In the empirical method, experts set a passing score for each item by judging every checklist item. The main subcategory of the empirical method is the Angoff method [6]. But the judges face the complexity of defining borderline group performance [7]. In the literature, borderline students are minimally competent students whose performance is at a level between pass and fail [8]. In the judgmental absolute method, also known as the borderline regression method (BRM), examiners put a global grade on each checklist in different OSCE stations such as history taking, physical examination, procedural skills, and health education [2].

Angoff method is based on judging individual checklist items, assuming that items are content-independent. Furthermore, it uses a hypothetical borderline group. This method has disadvantages of setting standards so high. Moreover, it is an expensive and complex method. In the borderline regression method, examiners focus on holistic clinical performance rather than individual item performance using global rating, then they regressed checklists’ score on global rating, finally, the results are used to determine checklist passing rate. The borderline regression method inherently has a reality check, as examiners observe students’ performance and assign them as borderline at the same time. The borderline regression method is feasible and gives more reliable results [8].

Furthermore, standard setting methods were classified as judgmental methods, empirical methods, and combination methods. In judgmental methods, judges inspect individual test items to judge how minimally competent person would perform on each item. On the other hand, in empirical methods, judges use the examination results to complete the standard setting process, where they use test data as part of standard-setting process. In combination methods, judges use both empirical and judgmental data [2]. Setting performance descriptors for borderline group is an essential step in all methods; however, it is a demanding cognitive task and may impair experts’ judgment [9]. 

We cannot ignore the subjectivity that judges face in all types and categories of standard settings [10]. As in some methods, experts estimate the probability of answering the test item by the borderline candidate. In other methods, judges should observe and evaluate students’ performance during the examination [9].

 Implementing the definition of minimally competent (borderline) group or student is difficult, although it might be seen as straightforward [11]. It is important to have a clear understanding of what is “borderline” group before the beginning of standard setting process. We can group consensus before the work by asking judges to describe borderline group based on their experience [10], or we can use a predetermined model for characteristics of borderline group performance in each OSCE station [12]. The performance of borderline group varies from one station to another depending on the nature of each station. This study aimed to examine the effect of using this predetermined model on the objectivity of standard setting method and to measure the reliability and quality metrics of OSCE.

## Materials and methods

Conceptual framework

**Figure 1:**
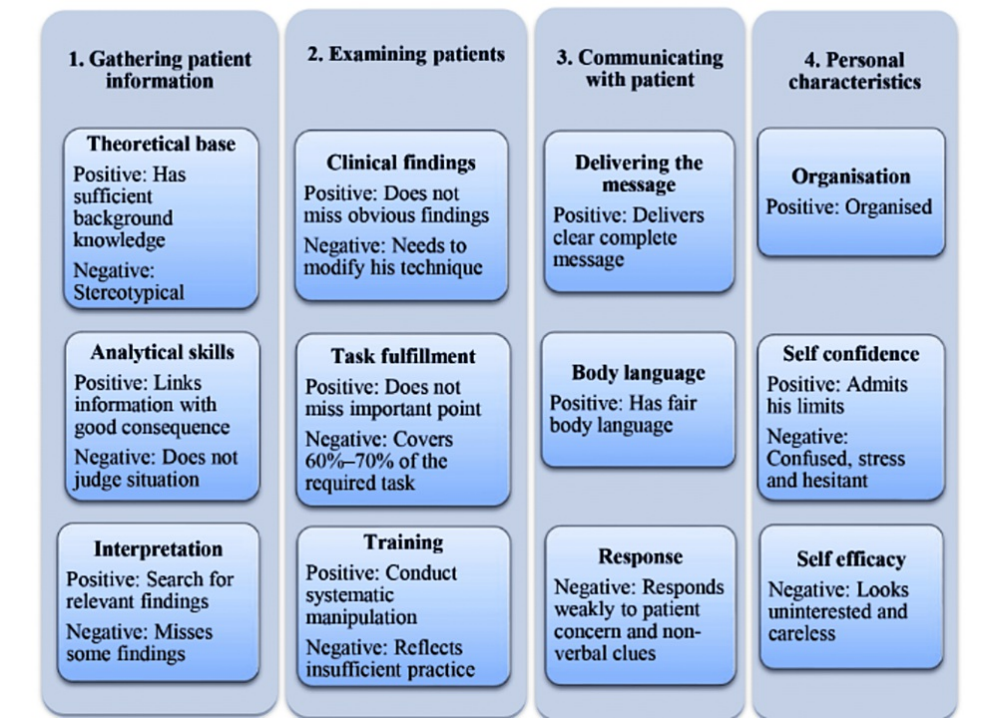
A model for characteristics of borderline group

This study aimed to assess the effect of using a model for minimally competent (borderline) group performance on the quality metrics of OSCE (Figure 1). Identification of borderline group is essential in many of the standard setting methods. It will affect the reliability and quality metrics of the OSCE results. Setting criteria by experts can help in the process of borderline group identification. We used this model with a group of examiners to test the effect of this model on OSCE reliability and quality metrics.

Study type, population, and sample

A quasi-experimental posttest-only design was utilized. Two groups of evaluators were investigated: one as experimental group and the other as control group. Twenty examiners who are experienced in teaching and assessment participated in the study. The intervention group was oriented about the used model before OSCE to test the effect of this model on their global scoring. On the other hand, the other group of examiners was not oriented about this model. Forty-one third-year medical students took part in a summative OSCE evaluating the gastrointestinal tract, cardiovascular and respiratory systems modules. Using two simultaneous circuits, students were assessed on the same day. Students were divided into two groups who were distributed among two mirrored circuits of OSCE stations. Each circuit consists of ten OSCE stations. Stations consist of four history taking, four examinations, one procedure, and one health education. Eight stations were patient encounters with standardized patient and one examiner. A manikin was used in the procedural station. Twenty stations were developed and distributed among two mirrored OSCE circuits, covering the respiratory system, cardiovascular, and gastroenterology.

Data collection tools and analysis

OSCE scoring consists of checklists that included 9-23 items for each station. To compute the cut scores, we used three-point scale (clear pass, borderline, and clear fail) for global rating. Data were analyzed using SPSS v. 22 (Armonk, NY: IBM Corp.). We used numbers and percentages for categorical variables, and means and standard deviations for continuous variables. We considered p< 0.5 as a cut score of statistical significance. We used borderline group method (BGM) to calculate the passing score for groups. The mean score of borderline groups was calculated for each OSCE station checklist, and then the mean of mean scores of all stations was considered as the cut score of the whole examination. We used Cronbach’s alpha to test internal consistency, Spearman correlation, and “alpha if item deleted” to measure quality metrics of OSCE for both circuits. And R2 coefficient was used to measure the squared linear correlation between the holistic rating score and the checklist score. An R2 = 0.5 is considered reasonable. The number of failures was estimated based on the cut score.

## Results

Table 1 shows the cut scores and reliability coefficients for both groups. The cut score of the first circuit, where we used the generated borderline students’ characteristics model, was 68% and the reliability of the results of this circuit was 0.85. While the cut score of the second circuit was 68.8% and the reliability was 0.57. It is evident that the reliability coefficient is considerably higher in the OSCE circuit where the developed model was used, and the cut scores are slightly different. The CVS passing score was the lowest passing score in both groups.

**Table 1:** Reliability statistics of the two OSCE circuits (41 students) OSCE: objective structured clinical examination

Circuit 1			Circuit 2		
Cronbach's alpha	Cronbach's alpha based on standardized items	No. of Items	Cronbach's alpha	Cronbach's alpha based on standardized items	No. of Items
0.856	0.847	10	0.574	0.650	10

Table 2 shows that if we delete item three, the reliability of OSCE will increase to a minimal degree in the circuit in which the model was used. While Table 3 shows that the reliability of OSCE will increase if we delete items three and 10 in the other circuit with a high degree.

**Table 2:** Internal consistency of circuit 1 OSCE stations OSCE: objective structured clinical examination

Circuit 1	Corrected item-total correlation	Squared multiple correlations	Alpha if item deleted
Station 1	0.667	0.842	0.833
Station 2	0.304	0.686	0.859
Station 3	0.008	0.538	0.875
Station 4	0.845	0.933	0.813
Station 5	0.587	0.826	0.840
Station 6	0.705	0.668	0.834
Station 7	0.682	0.812	0.834
Station 8	0.435	0.621	0.852
Station 9	0.753	0.859	0.829
Station 10	0.639	0.625	0.835

**Table 3:** Internal consistency of circuit 2 OSCE stations OSCE: objective structured clinical examination

Circuit 2	Corrected item-total correlation	Squared multiple correlations	Alpha if item deleted
Station 1	0.483	0.533	0.472
Station 2	0.340	0.481	0.547
Station 3	0.193	0.554	0.692
Station 4	0.605	0.633	0.436
Station 5	0.451	0.781	0.502
Station 6	0.367	0.638	0.522
Station 7	0.349	0.449	0.531
Station 8	0.535	0.727	0.511
Station 9	0.054	0.629	0.583
Station 10	0.011	0.527	0.694

Table 4 shows that R2 is more in most stations in circuit one, where the hypnotized model was used, except for stations 1 and 3 where R2 was better in circuit two. The number of failures was more in stations 1, 4, 5, 7, 8, and 10 in the first circuit.

**Table 4:** Correlation coefficient and number of failures for each circuit

Station no.	Circuit 1		Circuit 2	
	R2	Number of failures	R2	Number of failures
1	0.354	3	0.731	2
2	0.215	0	0.095	0
3	0.181	0	0.636	1
4	0.889	4	0.853	1
5	0.964	1	0.526	0
6	0.362	0	0.108	0
7	0.647	1	0.379	0
8	0.832	6	0.364	0
9	0.559	0	0.085	0
10	0.399	2	0.010	1

## Discussion

It is important to evaluate the assessment, evidence of validity of the examination scores are needed to ensure the quality of an OSCE [13]. Delivery of OSCEs requires large numbers of examiners, standardized patients, or simulators, and often students are distributed across parallel sites. This leads to difficulties with standardization, which will be subject to evaluators’ performance even with using checklist. Utilizing quality metrics effectively for OSCE is central [14].

Standard setting, or setting cut score, is an essential step to increase the validity of results obtained from OSCE. This is especially crucial in high stake examinations, where pass-fail decision has implications for learner, educator, and patient [15]. Standard setting requires clear conceptualization of borderline group performance, which is the most difficult and demanding task [9]. We used the hypothesized model for borderline group to measure the effect of having a performance descriptor of borderline group, beforehand, on quality metrics and reliability of OSCE [12].

The internal structure validity evidence is measured by Cronbach’s alpha analysis. The generated model for borderline students’ characteristics affected OSCE reliability as it was higher in the circuit in which the model was used. The two circuits of OSCE have the same number and type of stations. The accepted value for alpha especially in high stakes assessments is 0.7 or above [14]. The difference in reliability may be due to the used model or the difference in performance between the two groups of students or examiners. However, the two groups of examiners had the same training regarding the used checklist and global grading.

Alpha if item deleted scores for each station should all be lower than the overall alpha score. If we deleted item two, in the circuit where the model was used, the reliability of OSCE will increase to a minimal degree. While the reliability of OSCE will increase if we delete items three and ten in the other circuit. This may be due that these items are measuring different constructs, or they need to be modified, there are teaching problems for the item, or finally the assessors are assessing to a different standard. In such case, quality improvement is needed for this station [16].

Mean scores for the CVS were the lowest, and this was comparable with a study conducted by Dwivedi et al. in 2020 [17]. Authors attributed this difference to the fact that CVS checklists are more challenging and include more items assessing clinical reasoning skills.

The squared linear correlation (R2 coefficient) between the global rating score and the checklist score is expected to be positively correlated. R2 is considered reasonable when it equals or more than 0.5. In the first circuit, R2 in stations 1, 2, 3, 6, and 10 was lower than 0.5, while in stations 2, 6, 7, 8, 9, and 10, the R2 was lower than 0.5. This indicates a mismatch between the checklist (dependent) and the global rating (independent), this means that some students may get more marks from the checklist, but evaluators were not satisfied with their overall performance, thus the itemized checklist could be a poor marker of competence. As a result, careful design of the station and the checklist items are required to reflect students’ level of performance [13]. High failure rates were noticed in station eight in circuit one which was CVS auscultation. High failure rate requires revisiting the teaching process, as this highlighted teaching problems [14].

This study has some limitations. The sample size of students and stations and larger sample size may have a different impact on our results and interpretation. Moreover, it would be better if more evidence of validity were gathered such as intergrade discrimination, and between group variation to assess the effect of other errors of measurement related to examiners, venues, or exam structure. Further research is needed through implementing the hypothesized model for borderline group in different settings and group of students.

## Conclusions

Ensuring the validity and reliability of evidence of examination is essential, especially in high stake OSCE. Standard setting affects the sequential validity evidence of the exam. Defining borderline group performance is an essential step in all methods of standards setting. However, visualizing the borderline group performance is a demanding cognitive task especially when examiners have less experience. Having a model describing the performance of borderline group beforehand will facilitate the standard setting process. The used model improved the internal consistency of the OSCE and linear correlation between global scoring and checklist score. Meanwhile, the effect of the used model on other OSCE quality metrics needs more investigation.
